# Bowel sound in predicting the severity of acute pancreatitis: Protocol of a prospective, multi-center study

**DOI:** 10.1371/journal.pone.0358660

**Published:** 2026-10-13

**Authors:** Ziying Han, Jianing Li, Hemiao Xu, Fanyi Kong, Yonghao Chen, Yanna Cai, Qingwei Jiang, Di Shi, Dong Wu

**Affiliations:** 1 Department of Gastroenterology, State Key Laboratory of Complex Severe and Rare Diseases, Peking Union Medical College Hospital, Chinese Academy of Medical Sciences & Peking Union Medical College, Beijing, China; 2 Chinese Academy of Medical Sciences and Peking Union Medical College, Beijing, China; 3 Department of Emergency, State Key Laboratory of Complex Severe and Rare Diseases, Peking Union Medical College Hospital, Chinese Academy of Medical Sciences & Peking Union Medical College, Beijing, China; University of Diyala College of Medicine, IRAQ

## Abstract

**Background:**

Acute pancreatitis (AP) is a common condition, with approximately 20% of patients developing severe acute pancreatitis (SAP), which is associated with a poor prognosis. Early identification and timely intervention for SAP patients are essential for improving clinical outcomes. This study aims to investigate whether bowel sound (BS) monitoring could facilitate the early recognition of SAP in AP patients.

**Methods:**

This study is a prospective, multi-center prognostic study. We will consecutively recruit newly diagnosed AP patients within 48h of disease onset who have not developed SAP at enrollment from the emergency departments of three centers from 01/04/2024. Upon enrollment, each patient will undergo continuous bowel sound monitoring for at least 48 hours using standardized equipment and procedures. The primary outcome is the occurrence of SAP within the first 7 days of hospitalization. Collected bowel sound data will be analyzed using an automated algorithm to derive five bowel sound acoustic parameters: bowel sound occurrence rate, dominant frequency, duration, amplitude, and inter-bowel-sound interval. These parameters will be used as predictors of SAP. Bowel sound analysts will be blinded to patients’ clinical information and outcomes, and treating clinicians will not have access to the bowel sound analysis results during the study. We will calculate the receiver operating characteristic (ROC) curve and area under the curve (AUC) to evaluate their predictive performance.

**Discussion:**

This study explores bowel sound monitoring as a non-invasive, real-time tool for assessing AP severity. Early BS changes correlate with prognosis, offering continuous physiological insights complementary to traditional scores. Despite confounders like clinical interventions, preliminary data show BS attenuation in SAP. Standardized protocols and noise-cancellation algorithms enhance reliability. Mechanistically, BS reflects intestinal dysmotility, aiding early deterioration detection. Future work will validate BS-guided interventions to optimize AP management.

**Trial registration:**

ClinicalTrials.gov NCT06867120

Chinese Medical Research Registration and Filing Information System MR-11-25-024794

## Background

Acute pancreatitis (AP) is a common acute abdominal condition, with a global prevalence of approximately 30–40 cases per 100,000 annually, and its incidence continues to increase [1]. According to the 2012 Atlanta classification system, AP is categorized into mild acute pancreatitis (MAP), moderately severe acute pancreatitis (MSAP), and severe acute pancreatitis (SAP) [2]. Approximately 20% of AP patients develop SAP, which carries a mortality rate of 20–30% [3,4]. A large population study suggests that the 90-day post-discharge mortality rate for AP patients can still reach 3% [5]. Moreover, AP can lead to complications such as post-pancreatitis diabetes, exocrine dysfunction, and even chronic pancreatitis, significantly increasing the healthcare burden on patients with AP [3]. List of abbreviations in this manuscript is provided in S1 Table.

Recent studies have found that intestinal function plays a significant role in the development and progression of AP [6]. The intestines not only facilitate digestion and absorption but also serve as a barrier against pathogens and harmful substances. Intestinal motility is essential for both digestive and absorptive functions, and it may also influence the integrity of the intestinal barrier [7]. AP is often associated with lower intestinal motility, which can lead to intestinal obstruction. Studies have shown that intestinal permeability is compromised in AP patients, correlating with disease severity [8]. AP modeling significantly inhibits intestinal peristalsis and gastric emptying rates in mice [9]. The reduced intestinal motility in AP mice is significantly correlated with small intestinal bacterial overgrowth [10]. Intestinal motility dysfunction leads to increased intestinal permeability, negatively affecting the intestinal barrier function. Previous studies have found that indicators of intestinal permeability, such as serum D-lactate, endotoxin, diamine oxidase, and intestinal fatty acid-binding protein (I-FABP), can help predict the prognosis of AP patients. Lower I-FABP levels have demonstrated strong negative predictive value for pancreatic necrosis and disease severity in AP patient [11]. Moreover, early-stage gut microbiota dysbiosis in AP patients not only predicts mortality and length of hospital stay but is also linked to the occurrence of acute respiratory distress syndrome, intra-abdominal infections, and other complications during hospitalization [12–14]. Therefore, bowel sound intensity can be an objective measure of patient’s intestinal motility, reflecting intestinal function and assisting in the prognosis of AP patients.

Currently, the definition of organ failure in patients with AP primarily focuses on the respiratory, renal, and cardiovascular systems [4]. Due to the lack of objective evaluations of intestinal function, intestinal dysfunction in AP patients has not been incorporated into assessments of organ failure or evaluations of disease severity. Changes in bowel sounds are typically assessed through clinical auscultation, however, there is currently no real-time, dynamic method for monitoring intestinal function. Continuous monitoring of bowel sounds can objectively assess intestinal motility in AP patients, aiding in the evaluation of intestinal organ failure and even assisting in predicting the prognosis of AP.

Therefore, we implement a novel bowel sound monitoring system to enable long-term, real-time, dynamic tracking of bowel sounds changes in AP patients. Bowel sound data is collected through a Bluetooth system, enabling continuous monitoring of intestinal function [15]. The device has demonstrated impressive sensitivity and specificity, both exceeding 90%, could effectively distinguishing bowel sounds from environmental noise [16]. Recent advances in bowel sound monitoring devices further highlight the focus on technical optimization for clinical applicability [17]. Additionally, previous studies have shown that bowel sound monitoring can help diagnose irritable bowel syndrome, avoiding additional invasive tests in patients with functional disorders [18,19]. It can also help predict the risk of sepsis in critically ill patients in the intensive care unit (ICU) and facilitate the early identification of individuals at high risk [20].

This study aims to recruit AP patients within 48 hours of disease onset and to dynamically monitor bowel sounds changes. It will explore the correlation between bowel sounds and disease severity, as well as evaluate the role of bowel sounds in predicting AP prognosis and guiding treatment.

## Methods

### Study design

This is a prospective, multi-center prognostic study using bowel sound monitoring to predict early progression to SAP in AP patients. This manuscript describes the protocol of an ongoing prospective, multicenter observational study conducted from 01/04/2024–31/12/2027, with no results generated yet. Participant recruitment and data collection are expected to be completed by the end of 2027, and the study results are anticipated to be published by the end of 2028. All patients who fulfil the inclusion criteria and do not meet the exclusion criteria will be approached for participation. Written informed consent is obtained from all participants. The Peking Union Medical College Hospital’s Research Ethics Committee has approved this study (approval no.I-25PJ1400) and the study is registered at Clinicaltrials.gov (NCT06867120). We will disseminate our study results through academic conferences and peer-reviewed public journals. The study design is presented in Fig 1, while the subsequent sections provide a comprehensive and detailed description of our study. The reporting of this protocol follows the applicable TRIPOD+AI recommendations (S2 Table) [21].

**Fig 1:**
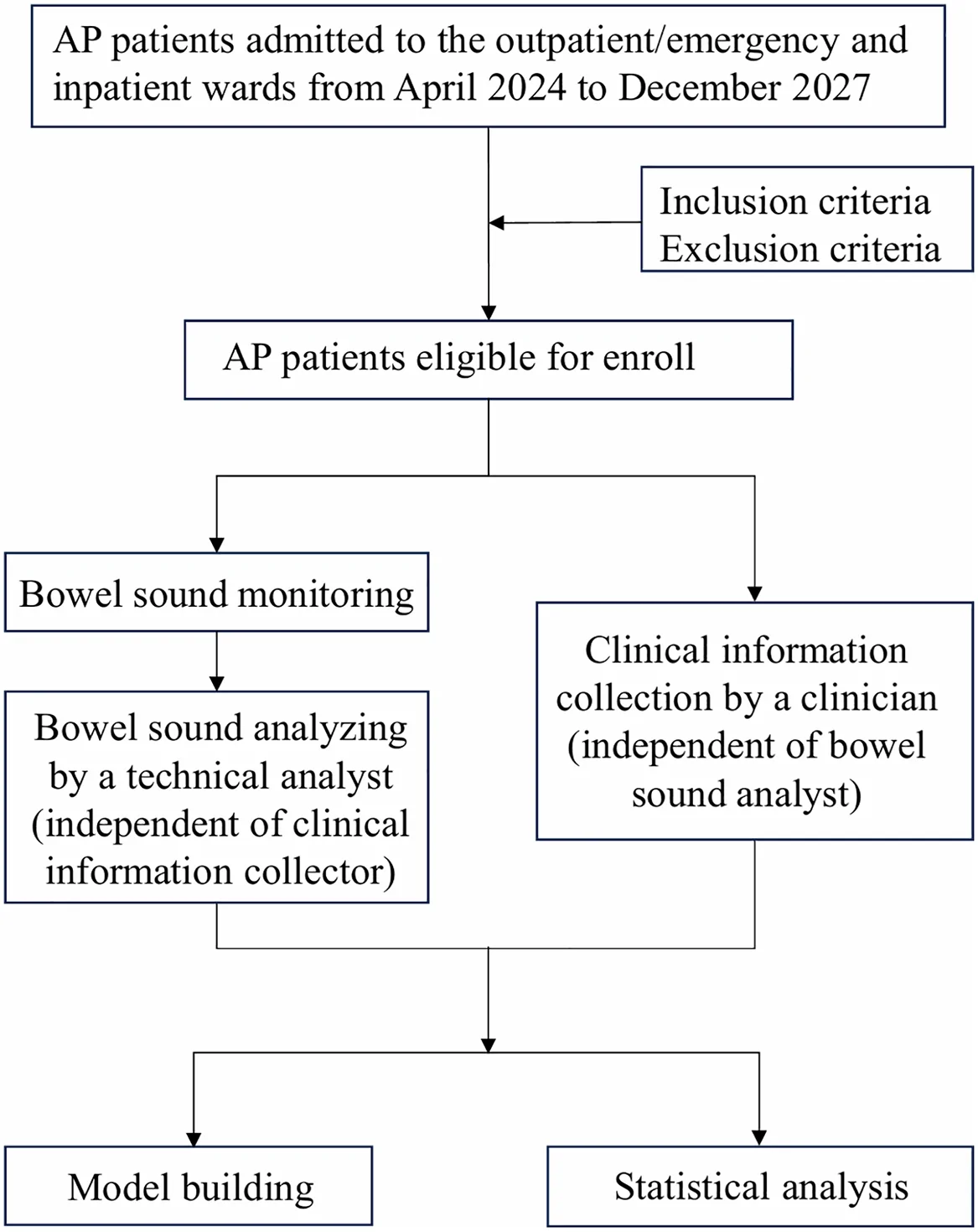
Flowchart of the study process.

### Study population

This study will include AP patients from Peking Union Medical College Hospital, Beijing Sixth Hospital, and Beijing Longfu Hospital from 01/04/2024–31/12/2027. At enrollment, the severity of AP could not be definitively determined in any patient.

#### Inclusion criteria.

1) Aged between 18 and 85 years old.
2) Meet the diagnostic criteria for AP, at least two of the following three: (a) characteristic abdominal pain; (b) serum amylase and lipase levels are greater than three times the upper limit of normal; (c) abdominal images with typical manifestations of AP.
3) AP patients within 48 hours of onset.
4) The patient or family members understand the study protocol and sign the informed consent form.

#### Exclusion criteria.

The patients who fulfill any of the following criteria will be excluded:

1) Patients with serious conditions requiring surgery and abdominal lavage.
2) Pregnant women.
3) The patients with muscle and nerve disorders (e.g., myopathy, myasthenia gravis, Parkinson’s disease), chronic pancreatitis, inflammatory bowel disease, cancer, and irritable bowel syndrome.
4) The patients with severe digestive, respiratory, cardiovascular, hematological, endocrine, psychiatric, and infectious diseases.
5) The patients have a history of abdominal surgery (except appendectomy or cholecystectomy).
6) Allergic to silicone, polycarbonate materials or medical plaster.

The intake of antibiotics, probiotics, and Chinese herbal medicine will be recorded before the collection of the swabs and will not be considered as exclusion criteria.

### Outcome measures

Among patients without SAP at enrollment, the primary outcome is whether AP patients develop SAP within the first 7 days of hospitalization. According to the revised Atlanta classification, MAP is characterized by the absence of organ failure and local or systemic complications; MSAP is characterized by transient organ failure resolving within 48 hours and/or local or systemic complications without persistent organ failure; and SAP is defined by persistent organ failure lasting more than 48 hours.

Secondary outcomes include in-hospital mortality, 90-day mortality, length of hospital stay, and organ failure. The adverse reaction evaluates the occurrence of cutaneous allergic reactions associated with the use of the bowel sound monitoring system**.** Post-discharge follow-up will be conducted to assess prognosis through outpatient visits or telephone interviews.

### Bowel sound monitoring

Upon securing informed consent, bowel sound monitoring will be initiated on the day of admission (Day 0) for patients who fulfill the inclusion criteria. The whole monitoring process is shown in Fig 2. A wearable monitoring sensor, which captures and records bowel sounds, will be affixed to the patient’s lower right abdomen by using medical-grade adhesives. The raw audio signals will be transmitted via Bluetooth to the receiver and subsequently relayed through a wireless network to a terminal for signal processing. This process includes recognition, extraction, and intelligent analysis of the data, which are converted into five bowel sound acoustic parameters: bowel sound occurrence rate, dominant frequency, duration, amplitude, and inter-bowel-sound interval. Bowel sound monitoring is planned to continue for more than 48 hours to assess their dynamic changes. At least 24 hours of valid bowel sound data will be required for model analysis. If monitoring is discontinued after at least 24 hours of valid recording, it will be flagged as premature termination but the available data will still be retained for model analysis. Patients with less than 24 hours of valid bowel sound recording will be excluded from the model analysis due to insufficient audio data. Nevertheless, these patients will be retained in the study flow records, and the number of exclusions and corresponding reasons will be reported.

**Fig 2:**
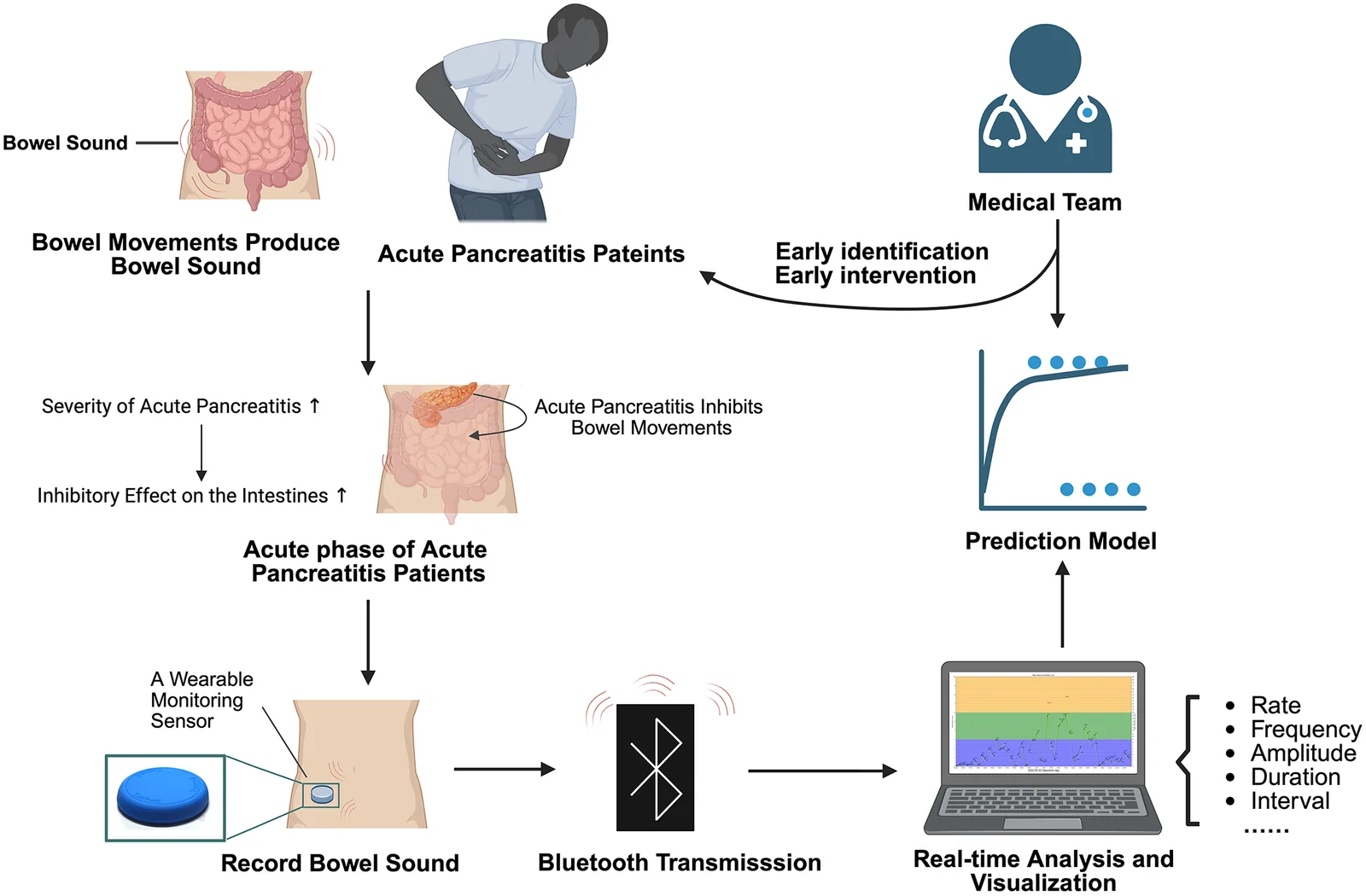
Schematic diagram of the placement of bowel sound monitoring patches and data transmission. The wearable monitoring sensor is placed on the right lower abdomen to continuously collect audio of the patient’s bowel sounds. The audio is transmitted to a computer via Bluetooth, and the software automatically analyzes it to generate visual images and objective data, which will be used to construct a prediction model. In the future, clinical physicians will use this prediction model to further assess the patient’s condition. During the present study, these results will not be provided to treating clinicians or used to guide clinical decision-making. The potential use of the model by clinicians represents an intended future application after its predictive performance has been adequately developed and validated.

Before study initiation, personnel responsible for bowel sound monitoring at all participating centers will receive standardized training. Identical monitoring devices, software versions, sensor placement procedures, and data acquisition protocols will be used across centers.

An independent blinding mechanism will be implemented. First, personnel responsible for bowel sound processing and feature extraction will be blinded to patients’ clinical information, treatments, and outcomes. Second, all clinicians will not have access to the bowel sound results during the study. Clinicians responsible for determining the occurrence of SAP will be blinded to the bowel sound results. All clinical assessments and treatment decisions will be based on conventional clinical information and current standards of care. Therefore, bowel sound monitoring results will not influence treatment decisions or patient outcomes during the present study. Bowel sound features and clinical outcomes will be linked using coded study identification numbers only after feature extraction and outcome assessment have been completed.

### Bowel sound analysis

The collected abdominal sound data were acquired at a sampling rate of 4 kHz with a 16-bit resolution. The recorded audio is divided into multiple consecutive 5-second segments without overlap [22]. To enable accurate identification of intestinal sounds, abdominal acoustic signals were collected and annotated from 20 healthy volunteers and 100 patients with various conditions, recruited from 6 medical centers under different environmental settings and time points, based on previous studies [23]. This process resulted in the construction of a dataset containing 17,605 annotated intestinal sound events, along with additional labels for other sound categories such as friction sounds, speech, and coughing. During the training phase, the recognition system extracted Mel-frequency cepstral coefficient (MFCC) features from the dataset and employed a convolutional recurrent neural network (CRNN) for classification and recognition.

The detailed analysis process includes the following steps:

**Step 1** The collected abdominal sound data were acquired at a sampling rate of 4 kHz with a 16-bit resolution.
**Step 2** Mel-frequency cepstral coefficient (MFCC) feature extraction is performed on each audio clip with a window length of 5 seconds and a hop length of 100 millisecond, generating an array of audio features [22].
**Step 3** Bowel sound event detection: The extracted feature arrays employed a convolutional recurrent neural network (CRNN) model for classification. The CRNN network is made up by a five-layer convolutional neural network (CNN), a bidirectional gated recurrent unit (BiGRU) network, and a fully connected layer. The model classified audio segments into different categories: bowel sounds, respiratory sounds, speech, friction sounds, coughs, snoring, and white noise. The output provided sound event labels with a 1-second temporal resolution for each 1-minute audio segment [23].
**Step 4** Bowel sound feature extraction: The bowel sound segments identified by the classification model within each 1-minute audio recording were extracted for feature calculation. Five bowel sound features were calculated, including rate, duration, amplitude, frequency, and interval. The bowel sound occurrence rate was defined as the number of bowel sound events per minute (events/min). Duration represented the length of each bowel sound event (s). Amplitude reflected the sound intensity (dB). Dominant frequency represented the characteristic frequency of bowel sounds (Hz). Inter-bowel-sound Interval was defined as the time between two consecutive bowel sound events (s).

Validation results demonstrated the model’s ability to effectively distinguish bowel sounds from environmental noise, achieving an average F1-score of 81.06%. In this research, abdominal sound recordings will be processed by the system to automatically identify bowel sound events and extract five bowel sound acoustic parameters for each patient: bowel sound occurrence rate, dominant frequency, duration, amplitude, and inter-bowel-sound interval. Audio recordings will undergo prespecified quality control before feature extraction. Recording segments affected by device disconnection, corrupted files, signal saturation, or excessive environmental noise that prevents reliable bowel sound identification will be classified as unusable and excluded. Valid monitoring duration will be calculated using only usable recording segments. Patients must have at least 24 hours of usable audio data to be included in the model analysis. The amount of excluded audio and the reasons for exclusion will be documented. Episodes of signal loss or sensor detachment, their duration, and their causes will be recorded. Missing audio segments will not be imputed. Bowel sound occurrence rates and other time-dependent parameters will be calculated using the valid recording duration as the denominator. The total valid monitoring duration, the proportion of unusable audio, and premature monitoring termination status will be included as technical covariates in sensitivity analyses to evaluate whether incomplete monitoring affects model performance.

### Audio data privacy and management

The terminal software will automatically process the recordings and identify audio segments containing bowel sound events. Audio segments dominated by environmental noise or speech will be excluded from subsequent analysis. Only audio segments identified as bowel sounds and the corresponding extracted acoustic parameters will be used in subsequent analyses. All audio data will be assigned a unique study identification number and stored on a secure, password-protected institutional server separately from personally identifiable information. Access to the audio data will be restricted to authorized members of the research team. Raw audio recordings that may contain environmental sounds or speech will not be publicly shared.

### Clinical information collection

Demographic data, including age, gender, and body mass index (BMI), as well as clinical symptoms such as abdominal distension, abdominal pain, bloody stools, constipation, and defecation patterns, will be recorded. Medical history will focus on conditions including hyperlipidemia and prior abdominal surgery. Laboratory assessments will include tests to evaluate hematological, hepatic, renal, and pancreatic function, along with C-reactive protein (CRP) levels. Diagnostic imaging will involve ultrasound and computed tomography (CT).

Disease severity will be assessed using a comprehensive approach with various scoring systems, including severity grading, Ranson score, modified Marshall score, Bedside Index for Severity in Acute Pancreatitis (BISAP) score, Acute Physiology and Chronic Health Evaluation II (APACHE II) score, and Acute Gastrointestinal Injury (AGI) score. Among these scoring systems, the Ranson and APACHE II scores will be used as established clinical predictors of SAP and compared with the bowel sound prediction models.

### Model building

The incidence is considered to be 20% based on previous literature and experiences from our hospital. Predictor variables include five bowel sound acoustic parameters: bowel sound occurrence rate, dominant frequency, duration, amplitude, and inter-bowel-sound interval. The primary analysis will construct prediction models using bowel sound acoustic parameters alone. The predictive performance of these models will be compared with that of the Ranson and APACHE II scores.

To evaluate whether bowel sound monitoring improves the predictive performance of these established scores, additional combined models will be constructed by adding bowel sound acoustic parameters to the Ranson and APACHE II scores. The area under the receiver operating characteristic curve (AUC) will be used to evaluate the predictive effect of these models. The minimum sample size required for model development was calculated using the pmsampsize package, following the method for developing clinical prediction models with binary outcomes proposed by Riley et al. [24]. The calculation assumed five candidate bowel sound predictor parameters, an anticipated SAP incidence of 20%, an expected C-statistic of 0.80, and a target global shrinkage factor of 0.90. Under these assumptions, at least 246 analyzable participants were required for model development. To preserve the minimum development sample size of 246 participants while allowing separate temporal validation, a prespecified 7:3 development-to-validation allocation will be used. Participants will be assigned chronologically according to their enrollment date: the first 246 analyzable participants will constitute the development cohort, and the subsequent 106 analyzable participants will constitute the temporal validation cohort. Allowing for an anticipated 10% attrition rate due to participant withdrawal, incomplete outcome data, or insufficient valid bowel sound recordings, the final recruitment target was increased to 392 participants.

The prediction models for the development of SAP within the first 7 days of hospitalization will be constructed in the development cohort using logistic regression (LR), random forests (RF), support vector machines (SVM), and K-nearest neighbors (KNN) in Python (version 3.12.2). Model development will include appropriate data preprocessing, handling of missing data, and hyperparameter tuning using cross-validation. If substantial class imbalance is observed, appropriate approaches, such as class weighting, will be considered. All model development procedures will be conducted using only the development cohort to minimize overfitting and prevent information leakage. The final models will then be evaluated in the temporal validation cohort.

Model performance will be evaluated in the temporal validation cohort in terms of discrimination, calibration, and clinical utility. Discrimination will primarily be assessed using the AUC. Where clinically appropriate, sensitivity, specificity, predictive values, and likelihood ratios will also be reported at clinically relevant thresholds. Calibration will be evaluated using calibration plots, calibration intercept and slope, and the Brier score. Clinical utility will be explored using decision curve analysis. The incremental value of bowel sound monitoring will be assessed by comparing the AUC of each clinical-score-only model and the corresponding combined model.

### Statistical analysis

This study will investigate the correlation about bowel sounds, clinical data and information related to their biospecimens in AP patients using Pearson correlation analysis.

Statistical analyses will be performed using SPSS version 26.0 and Python version 3.12.2. Continuous variables will be summarized as mean and standard deviation or median and interquartile range, as appropriate, and categorical variables as frequencies and percentages. Between-group comparisons will be conducted using appropriate parametric or nonparametric tests. Correlations between continuous variables will be assessed using Pearson’s or Spearman’s correlation coefficients, as appropriate. All statistical tests will be two-sided, with p < 0.05 considered statistically significant.

The extent and patterns of missing data will be summarized for each variable and handled using appropriate imputation methods. During model development, imputation and other preprocessing procedures will be performed within the training data to minimize information leakage. Potential center effects and data-quality-related factors will be considered in sensitivity analyses where appropriate.

The primary prediction models will focus on the five prespecified bowel sound acoustic parameters. Secondary analyses may incorporate relevant demographic and clinical variables, as well as established severity scores, to assess model robustness and incremental predictive value. The final selection and specification of predictors will be determined based on clinical relevance, data availability, and model performance while minimizing overfitting.

## Discussion

This study utilizes a novel bowel sound monitoring system that enables long-term data acquisition, offering a potential pathway for timely clinical assessments in AP. The correlation between bowel sound parameters and patient prognosis may provide valuable insights into dynamic changes occurring within the gastrointestinal system during AP.

This study aims to enroll AP patients within 48 hours of onset to capture early modifications in bowel sounds, which may serve as predictive indicators of disease severity. Previous studies suggest that serum biomarkers and intestinal microbiota are correlated with adverse outcomes of AP patients. However, sample collection and testing are both invasive and time-consuming. This study explores the correlation between bowel sounds and clinical outcomes in AP patients. As an efficient and non-invasive monitoring method, bowel sound monitoring can help determine the severity of AP.

Bowel sound monitoring may facilitate the early identification of patients at high risk of SAP by complementing established severity scores with continuous and objective information. This integration could enhance prognostic accuracy, inform decisions like ICU admission, and enable personalized nutrition management. For instance, guiding enteral feeding initiation or volume titration based on objective bowel sound recovery trends, potentially improving outcomes in AP and other critical gastrointestinal conditions. In the present study, bowel sound results will not be disclosed to treating clinicians or used to guide patient management. If the model demonstrates adequate predictive performance, its ability to support the early identification and treatment of patients at risk of SAP will be evaluated in future prospective interventional studies.

We acknowledge that clinical interventions such as analgesia, fluid resuscitation, and feeding may influence bowel sound patterns. Although these influences cannot be completely eliminated in routine clinical practice, our preliminary data demonstrate significant attenuation of bowel sounds in patients who subsequently develop MSAP or SAP. Besides, we will conduct subgroup analyses to validate the relationship between bowel sound and AP severity relationship across varying intervention levels. Regarding other confounding factors, we recognize that obesity may attenuate bowel sound amplitude, but other core metrics (e.g., frequency, duration) are less affected. What’s more, to enhance the generalizability of the experimental results, standardized protocols and centralized training will be employed to reduce variability across research centers, while device-integrated noise-cancellation algorithms will address issues of inconsistent environments across hospitals. The temporal validation cohort is expected to include approximately 21 SAP events. This relatively small number of events may limit the precision of estimates of model discrimination, calibration, and clinical net benefit. Therefore, all performance estimates will be reported with 95% confidence intervals, calibration and decision curve analyses will be interpreted cautiously, and further external validation in a larger independent cohort will be required.

Mechanistically, diminished bowel sound reflects intestinal dysmotility in SAP, driven by systemic inflammation and neural disruption. While precise acoustic feature-pathophysiology links require further study, bowel sound monitoring offers a unique objective tool quantifying this dysfunction. Rather than immediately replacing episodic scores, it provides continuous, real-time functional data that may facilitate earlier detection of deterioration or recovery.

In conclusion, this research endeavors to address the current deficiency in real-time intestinal function monitoring in AP. By exploring the prediction potential for disease severity of AP, this study may contribute to the formulation of treatment protocols and the enhancement of patient care. Future work will validate findings in larger cohorts, refine clinical algorithms, and prospectively evaluate bowel sound – guided interventions to optimize AP management protocols.

## Supporting information

S1 Table List of abbreviations. Abbreviations used in the main text and figures are listed alphabetically.
(DOCX)

S2 Table TRIPOD+AI checklist for the reporting of prediction model studies. Checklist items are reproduced from the official TRIPOD+AI checklist. D, model development; E, model performance evaluation. Items requiring study results are marked not applicable because this manuscript reports an ongoing protocol.
(DOCX)
