# Supplementary material for: Bowel sound in predicting the severity of acute pancreatitis: Protocol of a prospective, multi-center study
Source: PLoS One. 2026 Oct 13;21(10):e0358660. doi: 10.1371/journal.pone.0358660 (PMC13660877; doi:10.1371/journal.pone.0358660)
Supplement: Supplementary file 1 [file pone.0358660.s001.docx]

**Supplementary Table S1. List of abbreviations**

Abbreviations used in the main text and figures are listed alphabetically.

| **Abbreviation** | **Full term** |
| --- | --- |
| AGI | Acute Gastrointestinal Injury |
| AP | Acute pancreatitis |
| APACHE II | Acute Physiology and Chronic Health Evaluation II |
| AUC | Area under the receiver operating characteristic curve |
| BiGRU | Bidirectional gated recurrent unit |
| BISAP | Bedside Index for Severity in Acute Pancreatitis |
| BMI | Body mass index |
| BS | Bowel sound |
| CNN | Convolutional neural network |
| CRNN | Convolutional recurrent neural network |
| CRP | C-reactive protein |
| CT | Computed tomography |
| dB | Decibel |
| F1 score | Harmonic mean of precision and recall |
| Hz | Hertz |
| I-FABP | Intestinal fatty acid-binding protein |
| ICU | Intensive care unit |
| kHz | Kilohertz |
| KNN | K-nearest neighbors |
| LR | Logistic regression |
| MAP | Mild acute pancreatitis |
| MFCC | Mel-frequency cepstral coefficient |
| MSAP | Moderately severe acute pancreatitis |
| RF | Random forest |
| ROC | Receiver operating characteristic |
| SAP | Severe acute pancreatitis |
| SPSS | Statistical Package for the Social Sciences |
| SVM | Support vector machine |
