# Supplementary material for: Bowel sound in predicting the severity of acute pancreatitis: Protocol of a prospective, multi-center study
Source: PLoS One. 2026 Oct 13;21(10):e0358660. doi: 10.1371/journal.pone.0358660 (PMC13660877; doi:10.1371/journal.pone.0358660)
Supplement: Supplementary file 2 [file pone.0358660.s002.docx]

**Supplementary Table S2. TRIPOD+AI checklist for the reporting of prediction model studies**

Checklist items are reproduced from the official TRIPOD+AI checklist. D, model development; E, model performance evaluation. Locations refer to the line numbering in the revised marked-up manuscript. Items requiring study results are marked not applicable because this manuscript reports an ongoing protocol.

| **Item** | **Section/topic** | **Development/evaluation** | **Checklist item** | **Reported on page/line** |
| --- | --- | --- | --- | --- |
| **1** | Title | D;E | Identify the study as developing or evaluating the performance of a multivariable prediction model, the target population, and the outcome to be predicted | Lines 1-2. |
| **2** | Abstract | D;E | See TRIPOD+AI for Abstracts checklist | Lines 41-73. Results-related abstract elements are not applicable to this protocol. |
| **3a** | Background | D;E | Explain the healthcare context (including whether diagnostic or prognostic) and rationale for developing or evaluating the prediction model, including references to existing models | Lines 77-132. |
| **3b** | Background | D;E | Describe the target population and the intended purpose of the prediction model in the context of the care pathway, including its intended users (eg, healthcare professionals, patients, public) | Lines 77-132 and 138-151; intended future clinical use: Lines 229-238 and 404-414. |
| **3c** | Background | D;E | Describe any known health inequalities between sociodemographic groups | Not reported in the current protocol. |
| **4** | Objectives | D;E | Specify the study objectives, including whether the study describes the development or validation of a prediction model (or both) | Lines 42-60, 129-132, and 138-151. |
| **5a** | Data | D;E | Describe the sources of data separately for the development and evaluation datasets (eg, randomised trial, cohort, routine care or registry data), the rationale for using these data, and representativeness of the data | Prospective multicentre cohort: Lines 47-60 and 138-155; development/evaluation allocation: Lines 341-357. |
| **5b** | Data | D;E | Specify the dates of the collected participant data, including start and end of participant accrual; and, if applicable, end of follow-up | Lines 50, 68-73, 140-143, and 153-155. |
| **6a** | Participants | D;E | Specify key elements of the study setting (eg, primary care, secondary care, general population) including the number and location of centres | Lines 47-50 and 152-155. |
| **6b** | Participants | D;E | Describe the eligibility criteria for study participants | Lines 157-180. |
| **6c** | Participants | D;E | Give details of any treatments received, and how they were handled during model development or evaluation, if relevant | Potential effects of analgesia, fluid resuscitation, and feeding are discussed at Lines 415-425; subgroup analyses are planned. |
| **7** | Data preparation | D;E | Describe any data pre-processing and quality checking, including whether this was similar across relevant sociodemographic groups | Audio processing and quality control: Lines 196-216 and 240-305; clinical-data preprocessing: Lines 351-356 and 371-382. |
| **8a** | Outcome | D;E | Clearly define the outcome that is being predicted and the time horizon, including how and when assessed, the rationale for choosing this outcome, and whether the method of outcome assessment is consistent across sociodemographic groups | Lines 52, 182-193, and 348-365. |
| **8b** | Outcome | D;E | If outcome assessment requires subjective interpretation, describe the qualifications and demographic characteristics of the outcome assessors | Not applicable as written: SAP is defined using the revised Atlanta classification (Lines 183-188). Assessor qualifications are not reported. |
| **8c** | Outcome | D;E | Report any actions to blind assessment of the outcome to be predicted | Lines 217-227. |
| **9a** | Predictors | D | Describe the choice of initial predictors (eg, literature, previous models, all available predictors) and any pre-selection of predictors before model building | Five prespecified acoustic predictors: Lines 267-281 and 324-340. |
| **9b** | Predictors | D;E | Clearly define all predictors, including how and when they were measured (and any actions to blind assessment of predictors for the outcome and other predictors) | Measurement and definitions: Lines 196-212 and 240-294; blinding: Lines 217-227. |
| **9c** | Predictors | D;E | If predictor measurement requires subjective interpretation, describe the qualifications and demographic characteristics of the predictor assessors | Largely not applicable: acoustic features are generated by an automated CRNN-based pipeline (Lines 240-294); technical personnel are blinded (Lines 217-227). |
| **10** | Sample size | D;E | Explain how the study size was arrived at (separately for development and evaluation), and justify that the study size was sufficient to answer the research question. Include details of any sample size calculation | Lines 330-347 and 426-431. |
| **11** | Missing data | D;E | Describe how missing data were handled. Provide reasons for omitting any data | Audio data: Lines 276-294; clinical data: Lines 351-356 and 378-382. |
| **12a** | Analytical methods | D | Describe how the data were used (eg, for development and evaluation of model performance) in the analysis, including whether the data were partitioned, considering any sample size requirements | Lines 340-357. |
| **12b** | Analytical methods | D | Depending on the type of model, describe how predictors were handled in the analyses (functional form, rescaling, transformation, or any standardisation) | Acoustic preprocessing and feature derivation: Lines 240-281. Further model-specific handling is not fully prespecified. |
| **12c** | Analytical methods | D | Specify the type of model, rationale†, all model building steps, including any hyperparameter tuning, and method for internal validation | Lines 348-357. Candidate methods and cross-validation are specified; model-specific rationale is not fully reported. |
| **12d** | Analytical methods | D;E | Describe if and how any heterogeneity in estimates of model parameter values and model performance was handled and quantified across clusters (eg, hospitals, countries). See TRIPOD-Cluster for additional considerations‡ | Standardisation: Lines 213-216 and 423-426; centre effects in sensitivity analyses: Lines 378-382. |
| **12e** | Analytical methods | D;E | Specify all measures and plots used (and their rationale) to evaluate model performance (eg, discrimination, calibration, clinical utility) and, if relevant, to compare multiple models | Lines 358-365 and 426-431. |
| **12f** | Analytical methods | E | Describe any model updating (eg, recalibration) arising from the model evaluation, either overall or for particular sociodemographic groups or settings | Not applicable to the current protocol; no model has yet been developed or evaluated. |
| **12g** | Analytical methods | E | For model evaluation, describe how the model predictions were calculated (eg, formula, code, object, application programming interface) | Not applicable at protocol stage; the final model has not yet been developed. |
| **13** | Class imbalance | D;E | If class imbalance methods were used, state why and how this was done, and any subsequent methods to recalibrate the model or the model predictions | Lines 353-354. Class weighting will be considered if substantial imbalance is observed. |
| **14** | Fairness | D;E | Describe any approaches that were used to address model fairness and their rationale | Not reported in the current protocol. |
| **15** | Model output | D | Specify the output of the prediction model (eg, probabilities, classification). Provide details and rationale for any classification and how the thresholds were identified | Partially reported at Lines 358-365; clinically relevant thresholds are mentioned, but the output and threshold-selection procedure are not fully prespecified. |
| **16** | Training versus evaluation | D;E | Identify any differences between the development and evaluation data in healthcare setting, eligibility criteria, outcome, and predictors | Lines 341-357. Cohorts use the same setting, eligibility, outcome, and predictors and differ by chronological enrollment period. |
| **17** | Ethical approval | D;E | Name the institutional research board or ethics committee that approved the study and describe the participant informed consent or the ethics committee waiver of informed consent | Lines 145-147 and 445-450. |
| **18a** | Funding | D;E | Give the source of funding and the role of the funders for the present study | Funding sources: Lines 462-468. The role of funders is not stated. |
| **18b** | Conflicts of interest | D;E | Declare any conflicts of interest and financial disclosures for all authors | Lines 458-461. |
| **18c** | Protocol | D;E | Indicate where the study protocol can be accessed or state that a protocol was not prepared | This manuscript is the study protocol (Lines 138-143); registry information is provided at Lines 68-73 and 146-147. |
| **18d** | Registration | D;E | Provide registration information for the study, including register name and registration number, or state that the study was not registered | Lines 68-73 and 146-147. |
| **18e** | Data sharing | D;E | Provide details of the availability of the study data | Lines 296-305 and 453-457. |
| **18f** | Code sharing | D;E | Provide details of the availability of the analytical code§ | Not reported in the current protocol. |
| **19** | Patient and public involvement | D;E | Provide details of any patient and public involvement during the design, conduct, reporting, interpretation, or dissemination of the study or state no involvement | Not reported in the current protocol. |
| **20a** | Participants | D;E | Describe the flow of participants through the study, including the number of participants with and without the outcome and, if applicable, a summary of the follow-up time. A diagram may be helpful | Not applicable: protocol; recruitment is ongoing and no results have been generated. Planned flow is shown in Figure 1. |
| **20b** | Participants | D;E | Report the characteristics overall and, where applicable, for each data source or setting, including the key dates, key predictors (including demographics), treatments received, sample size, number of outcome events, follow-up time, and amount of missing data. A table may be helpful. Report any differences across key demographic groups | Not applicable: protocol; no results have been generated. |
| **20c** | Participants | E | For model evaluation, show a comparison with the development data of the distribution of important predictors (demographics, predictors, and outcome) | Not applicable: protocol; no model evaluation results are available. |
| **21** | Model development | D;E | Specify the number of participants and outcome events in each analysis (eg, for model development, hyperparameter tuning, model evaluation) | Not applicable to results. Planned analyzable sample sizes are reported at Lines 334-347. |
| **22** | Model specification | D | Provide details of the full prediction model (eg, formula, code, object, application programming interface) to allow predictions in new individuals and to enable third party evaluation and implementation, including any restrictions to access or reuse (eg, freely available, proprietary)¶ | Not applicable: protocol; the final model has not yet been developed. |
| **23a** | Model performance | D;E | Report model performance estimates with confidence intervals, including for any key subgroups (eg, sociodemographic). Consider plots to aid presentation | Not applicable to results. Planned measures and 95% confidence intervals are described at Lines 358-365 and 426-431. |
| **23b** | Model performance | D;E | If examined, report results of any heterogeneity in model performance across clusters. See TRIPOD-Cluster for additional details‡ | Not applicable to results. Centre effects are planned for sensitivity analyses (Lines 378-382). |
| **24** | Model updating | E | Report the results from any model updating, including the updated model and subsequent performance | Not applicable: protocol; no model updating has been performed. |
| **25** | Interpretation | D;E | Give an overall interpretation of the main results, including issues of fairness in the context of the objectives and previous studies | Not applicable to results. Anticipated clinical relevance is discussed at Lines 391-414. |
| **26** | Limitations | D;E | Discuss any limitations of the study (such as a non-representative sample, sample size, overfitting, missing data) and their effects on any biases, statistical uncertainty, and generalisability | Lines 415-431. |
| **27a** | Usability of the model in the context of current care | D | Describe how poor quality or unavailable input data (eg, predictor values) should be assessed and handled when implementing the prediction model | Audio-quality and missing-input procedures: Lines 276-294. Clinical implementation has not yet been evaluated. |
| **27b** | Usability of the model in the context of current care | D | Specify whether users will be required to interact in the handling of the input data or use of the model, and what level of expertise is required of users | Monitoring personnel training: Lines 213-216; intended future clinician use: Lines 229-238 and 404-414. |
| **27c** | Usability of the model in the context of current care | D;E | Discuss any next steps for future research, with a specific view to applicability and generalisability of the model | Lines 404-414, 426-443. |

*Checklist source: Collins GS, Moons KGM, Dhiman P, et al. TRIPOD+AI statement: updated guidance for reporting clinical prediction models that use regression or machine learning methods. BMJ. 2024;385:e078378. doi:10.1136/bmj-2023-078378.*

*D=items relevant only to the development of a prediction model; E=items relating solely to the evaluation of a prediction model; D;E=items applicable to both. †Separately for all model building approaches. ‡See TRIPOD-Cluster. §Analytical code includes data cleaning, feature engineering, model building, and evaluation. ¶Implementation code produces an estimate of risk for a new individual.
